# Predatory publications in evidence syntheses

**DOI:** 10.5195/jmla.2019.491

**Published:** 2019-01-01

**Authors:** Amanda Ross-White, Christina M. Godfrey, Kimberley A. Sears, Rosemary Wilson

**Affiliations:** Associate Librarian, Bracken Health Sciences Library, Queen’s University, Kingston, ON, Canada, amanda.ross-white@queensu.ca; Associate Professor, School of Nursing, Queen’s Joanna Briggs Collaboration, Queen’s University, Kingston, ON, Canada, christina.godfrey@queensu.ca; Associate Professor and Associate Director (Healthcare Quality), School of Nursing, Queen’s University, Kingston, ON, Canada, kim.sears@queensu.ca; Associate Professor, School of Nursing, Queen’s University, Kingston, ON, Canada, rosemary.wilson@queensu.ca

## Abstract

**Objectives:**

The number of predatory journals is increasing in the scholarly communication realm. These journals use questionable business practices, minimal or no peer review, or limited editorial oversight and, thus, publish articles below a minimally accepted standard of quality. These publications have the potential to alter the results of knowledge syntheses. The objective of this study was to determine the degree to which articles published by a major predatory publisher in the health and biomedical sciences are cited in systematic reviews.

**Methods:**

The authors downloaded citations of articles published by a known predatory publisher. Using forward reference searching in Google Scholar, we examined whether these publications were cited in systematic reviews.

**Results:**

The selected predatory publisher published 459 journals in the health and biomedical sciences. Sixty-two of these journal titles had published a total of 120 articles that were cited by at least 1 systematic review, with a total of 157 systematic reviews citing an article from 1 of these predatory journals.

**Discussion:**

Systematic review authors should be vigilant for predatory journals that can appear to be legitimate. To reduce the risk of including articles from predatory journals in knowledge syntheses, systematic reviewers should use a checklist to ensure a measure of quality control for included papers and be aware that Google Scholar and PubMed do not provide the same level of quality control as other bibliographic databases.

## INTRODUCTION

Since librarian Jeffery Beall first coined the term “predatory publishers” in 2010, listing nine journal publishers he believed engaged in questionable practices, the numbers of questionable academic publications have increased considerably [1]. Despite being explicit that he used the term “predatory” with caution, it is the name most frequently used to describe a nebulous concept of research journal publishers who use unethical business practices, minimal or no peer review, or limited editorial oversight to publish articles that are below a minimally accepted standard of quality [2]. In 2012, Beall began publishing regular blog postings on journals and publishers he deemed predatory, a list of journals to exclude that quickly grew in size, commonly called “Beall’s list.” Although he shut down the blog in January 2017, several archived versions are available.

There are no generally accepted criteria of what makes a journal predatory. This can pose a problem as both Beall’s list and its various replacements are not always clear in their definition of what makes a journal predatory or what a specific journal or publisher has done to gain entry to the list [3]. Predatory journals are often, but not exclusively, linked to open access publishing models, and an earlier reference to the proliferation of these journals linked the gold open access model of publishing to the rise in these questionable journals [4].

These journals are increasing rapidly in number and have the potential to alter the results of research syntheses. It is unknown how often predatory publications are cited in systematic reviews and other research syntheses. If the open access model used by many of these predatory publications makes them more accessible to systematic reviewers and their low standards lead to the publication of flawed or fraudulent research, future research could be compromised by prior errors. The authors sought to determine the degree to which articles from predatory publications are cited in systematic reviews.

## METHODS

As there is no consensus on criteria or strict definition of predatory journals, we selected a list of health and biomedical sciences journals from a publisher considered by many to be predatory, including the US Federal Trade Commission [5–8]. This journal publisher was chosen for two reasons: it was included on the now-defunct Beall’s list and legal action was taken against it by the US Federal Trade Commission [9]. From the list of journal titles on this publisher’s website, which numbered over 1,000, we created a list of 459 journals with health and biomedical sciences titles.

Using information from Google Scholar, we searched each predatory journal title individually and downloaded citations of articles published in these journals into EndNote x7 citation management software. We then used Google Scholar’s forward reference searching feature to determine whether the articles were cited in systematic reviews or meta-analyses. Only articles that used the words “systematic review” or “meta-analysis” in the title or clearly indicated in the abstract that a systematic process was used in the review methodology were included. Google Scholar was chosen to determine the number of citations of the indexed papers and to identify the citing systematic reviews, because most predatory journals are not indexed in traditional bibliometric databases and can only be found using Google and Google Scholar. Both index articles and citing reviews are available as an Excel spreadsheet in our institutional repository <http://dx.doi.org/10.5683/SP2/VMMBNH>. Searches were conducted from June to August 2017.

## RESULTS

Of the 459 journals investigated, 145 (31.6%) appeared to have never published any articles but to exist in title only. A search of the publisher’s websites revealed no papers published in these 145 journals but only solicitations for submissions. Sixty-four journals (13.9%) had published at least 1 article, but none of these articles had been cited by another publication, peer-reviewed or otherwise. The remaining 250 journals (54.5%) had 6,302 articles that had been cited at least once. Of these, 120 articles were cited by at least 1 systematic review. These 120 articles were published in 62 unique journals. As each systematic review could cite more than 1 article, a total of 157 systematic reviews cited an article from a journal from this predatory publisher. Of these 157 systematic reviews that cited a paper from a predatory journal, only 16 were themselves published in a predatory journal, whereas 4 were book chapters or doctoral dissertations and 137 were published in journals believed to be reputable and published by major international publishers (e.g., Wiley, Springer, Elsevier, Taylor & Francis, Sage).

Of the 459 journal titles from our initial list, only 1 title was indexed in MEDLINE. Another 7 were indexed in Embase, and 2 were indexed in CINAHL. Nine of the 10 journal titles indexed in bibliographic databases were journals that had previously been published by reputable scientific organizations that had been bought by the predatory publisher after the decision to index the journals [5, 6]. In addition, 39 of the journals with articles cited in a systematic review or meta-analysis had select publications in PubMed Central (PMC) in compliance with public access policies requiring authors of National Institutes of Health–funded research to deposit completed manuscripts in PMC.

## DISCUSSION

The purpose of peer review is to establish and maintain a standard for research in a particular field. This standard not only keeps researchers honest, but also allows readers (or authors of a systematic review) to feel secure that the rigor of the research and interpretation of the findings has been addressed and maintained by the publishing journal and that the article can be considered to make a valuable contribution to the state of knowledge. Given that not all readers are experts in content or methodology, the contribution of a manuscript without the standards provided by peer review is suspect at best.

The publisher we selected for this examination is allegedly engaged in questionable business practices and is known to have limited, or even nonexistent, peer review. In particular, the publisher has been found in a US federal court injunction to not engage in peer review, to fraudulently misrepresent their impact factor, and to engage in deception by failing to adequately disclose publication fees [9]. For this reason, we are concerned to see continued citing of research published in these journals, particularly in research syntheses, without knowing whether the authors of the reviews are aware of this problematic history of the journals.

Systematic review authors need to be vigilant for articles published in predatory journals that can appear to be legitimate. Using a checklist or critical appraisal tool to ensure that only papers of high quality are included in a research synthesis will reduce the risk of including poor-quality research and potentially changing outcomes on that basis. This is the case for both poor-quality research published in high-quality journals and poor-quality research published in predatory journals. A plethora of critical appraisal tools are available for evaluating research by employing many different research methodologies [10–13]. Systematic review publishers such as Cochrane, Campbell, the Evidence for Policy and Practice Information (EPPI), and the Joanna Briggs Institute all recommend the use of critical appraisal tools to evaluate the quality of research cited in a systematic review [14, 15].

While the criteria for what makes a publication predatory are fluid, there are some options for both researchers publishing articles and authors of knowledge syntheses for checking the validity of publications. This list of options is by no means exhaustive and is subject to change:

- Check to see if the publisher is a member of reputable publishing organizations, of which some examples are World Association of Medical Editors (WAME), Committee on Publication Ethics (COPE), and the International Academy of Nursing Editors (INANE) [16].
- Confirm that journal websites contain accurate and current information from an independent source (e.g., if the website indicates that the journal is indexed in PubMed, check MEDLINE to confirm).
- Consult Jeffery Beall’s original published criteria [17], or use Think. Check. Submit.

To reduce the likelihood that systematic reviewers engage with predatory publications, the producers of bibliographic databases—including MEDLINE, Embase, and CINAHL—should maintain awareness of the changing ownership of journals, as nine of the ten predatory journals indexed in bibliographic databases in this study were formerly legitimate journals that had been sold to a predatory publisher [5]. To further limit the impact on research syntheses of flawed and potentially fraudulent research, awareness on the part of researchers that Google Scholar and PubMed do not provide the same level of quality control as other bibliographic databases is critical. Authors may have themselves added the articles to PubMed Central under the public access policy.

Other researchers have found similar instances in which predatory journals were being included in high-quality bibliographic databases [18]. Researchers also need to be aware of the differences between PubMed and MEDLINE and what it means for a journal to be indexed in MEDLINE as opposed to appearing in PubMed, in some instances due to author-submitted manuscripts.

It is also important to note that not everything published in a predatory journal is fraudulent or of poor quality. Ethical researchers can also be caught in the predatory trap. It is also true that many systematic reviews do not limit their inclusion criteria to the peer-reviewed literature, including, for example, grey literature. However, if systematic review authors include predatory publications on the presumption of peer review, this poses a problem. Future research should evaluate the quality of the indexed papers using validated tools. It is unknown what percentage of articles in predatory publications is of insufficient quality to be published elsewhere. We also encourage PMC to ensure the quality of author-supplied submissions.

When systematic reviewers are searching for studies, they should exercise caution when an article is only found in Google Scholar and not through a search of bibliographic databases. This can be an indication that either the search in bibliographic databases needs improvement (because known indexed articles are not being found) or the articles may be in a predatory journal that is not included in reputable bibliographic databases. Other researchers have similarly expressed caution about the use of Google Scholar as a source in systematic reviews and meta-analyses [19, 20], as well as its use as a research tool in general [21, 22]. As only 10 of the 459 investigated journals were indexed in bibliographic databases—9 of which because they were small society publishers that had been purchased by a larger predatory organization—it would appear that these predatory publications are consistently failing to meet indexing criteria for these databases. While there may be a myriad of reasons that legitimate research is not indexed by any given bibliographic database, such as scope of the research or publication date, a failure to meet the criteria of all of the most significant bibliographic databases should raise some concerns.

Both bibliographic databases and journals are in a continual state of change, so these results are not generalizable and subject to change in future. However, predatory publications are not likely to disappear any time soon. Systematic reviewers must be cautious both in evaluating research by carefully considering its source and in seeking publication themselves.
